# Sensorimotor Alterations Induced by Novel Fentanyl Analogs in Mice: Possible Impact on Human Driving Performances

**DOI:** 10.2174/1570159X21666221116160032

**Published:** 2023-01-01

**Authors:** Sabrine Bilel, Arianna Giorgetti, Micaela Tirri, Raffaella Arfè, Virginia Cristofori, Beatrice Marchetti, Giorgia Corli, Lorenzo Caruso, Giorgio Zauli, Raffaele Giorgetti, Matteo Marti

**Affiliations:** 1 Department of Translational Medicine, Section of Legal Medicine and LTTA Center, University of Ferrara , 44121 Ferrara, Italy;; 2 Department of Medical and Surgical Sciences, Unit of Legal Medicine, University of Bologna, Via Irnerio 49, 40126 Bologna, Italy;; 3 Department of Chemistry and Pharmaceutical Sciences, University of Ferrara, Italy;; 4 Department of Environment and Prevention Sciences, University of Ferrara, Ferrara, Italy;; 5 Research Department, King Khaled Eye Specialistic Hospital, Riyadh, Saudi Arabia;; 6 Department of Excellence of Biomedical Science and Public Health, Faculty of Medicine, Polytechnic University of Marche, Ancona, Italy;; 7 Collaborative Center of the National Early Warning System, Department for Anti-Drug Policies, Presidency of the Council of Ministers, Ferrara, Italy

**Keywords:** Acrylfentanyl, Furanylfentanyl, Ocfentanyl, naloxone, sensorimotor alterations, novel psychoactive substances, DUID, opioids

## Abstract

Operating a vehicle is a complex task that requires multiple cognitive functions and psychomotor skills to cooperate. Driving might be impaired by licit or illicit drugs, including novel psychoactive substances (NPS) and novel synthetic opioids (NSO), the effects of which are still yet to be elucidated in humans. In the present work, a revision of the literature regarding the psychomotor impairing effects of Fentanyl (FENT) and three analogues (Acrylfentanyl, Ocfentanyl and Furanylfentanyl) is presented, as emerged by experimental studies on humans, driving under the influence of a drug (DUID) and intoxication cases. An experimental study on a mouse model evaluated the sensorimotor alterations induced by FENT and the three fentalogs. Acute systemic administration of the four opioids (0.01-15 mg/kg i.p.) dose-dependently decreased the visual object and placing tests, the acoustic and the tactile responses of mice. The preclinical data are in accordance with the data that emerged from the revision of the literature regarding experimental data on humans, driving under the influence of drugs and intoxication cases, suggesting that novel synthetic opioids might affect the psychomotor performances on daily human tasks with a particular focus on driving.

## 1 INTRODUCTION

Driving under the influence of drugs (DUID) refers to the act of operating a vehicle following ingestion, inhalation, absorption, or injection of drugs or medications other than alcohol, that could interfere with the capacity to drive an automobile safely [1]. Driving is a complex task, where the driver continuously elaborates and responds to information received from the external surroundings, and requires several cognitive and psychomotor functions to cooperate [2]. Many substances, both licit and illicit, may cause impairment of driving performance, affecting the body and the behavior in different ways. The most reported effects in cases of DUID consist of impairments of psychomotor skills and cognitive functions critical to driving, including vigilance, time and distance perception and monitoring, visual acuity, cognition, judgement and risk-taking behavior, reaction time, divided attention, keeping co-ordination and balance [2, 3]. A great alarm has been raised recently by the increase of DUID and traffic accidents due to the use of drugs. Cannabis was the illicit drug most frequently detected in cases of DUID, followed by cocaine while amphetamines and illicit opioids were less frequently detected. In addition to traditional substances, novel psychoactive substances (NPS) have also been related to DUID cases. Over the period from January 2019 to April 2020, 670 toxicology cases involving 46 individual NPS were reported to the UNODC. Of these cases, 62% were classified as DUID [4]. Synthetic cathinones are the most frequently detected NPS in Europe together with synthetic cannabinoids. However, the reports regarding the involvement of other NPS in DUID cases are very limited. Many NPS cannot be detected in road and toxicological tests and that could be a good reason for a driver to consume NPS rather than traditional compounds [5].

**Fig. (1):**
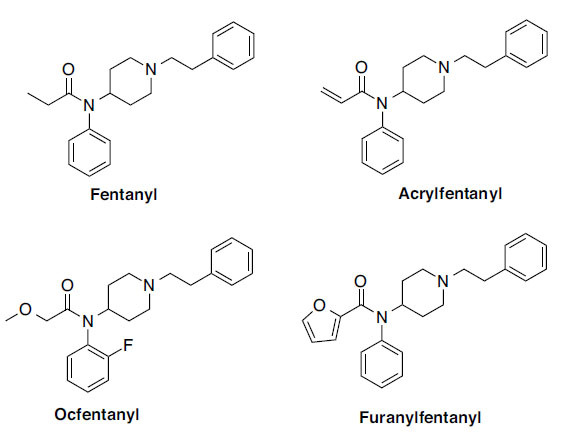
Chemical structures of Fentanyl; Acrylfentanyl; Ocfentanyl and Furanylfentanyl.

Novel Synthetic Opioids (NSO) is a growing class of NPS that consists of 67 compounds monitored by the European Monitoring Centre for Drug and Drug Addiction (EMCDDA) from 2009 and 2020, including 10 molecules that emerged just in 2020 [6]. Opioids, particularly in the setting of non-therapeutic consumption, have been reported to impair cognitive function, induce drowsiness, and increase crash risk [7]; however, little is known regarding the effects of NSO on drivers and psychomotor performances relevant for operating a vehicle. The highly potent synthetic fentanyl have also been reported in cases of DUID [3]. These substances are structurally and pharmacologically related to fentanyl (FENT) with some substitutions. Fentanyl derivatives (FENS) are sold as fentanyl substitutes, as heroin, and as contaminants in counterfeit prescription drugs. Among them, Acrylfentanyl (ACRYLF), Ocfentanyl (OCF), and Furanylfentanyl (FUF; Fig. **1**) have been found in cases of DUID in Europe [3].

The present study showcasesa literature review regarding the psychomotor effects connected to ACRYLF, OCF, FUF and FENT, together with experimental data on animals administered the same compounds.

## 2 PSYCHOMOTOR PERFORMANCES IN HUMANS - LITERATURE REVIEW

The study of psychomotor performances in humans is strongly affected on one side by ethical limitations in conducting experiments and on the other side by the biases connected to the interpretation of case reports, case studies and questionnaires. Due to the limited sample, experimental data are mostly lacking, and it is difficult to draw some scientific conclusions from case studies and case reports. Questionnaires and emergency departments evaluation might be only based on self-reported doses and symptoms, in the absence of forensic proof or analytical confirmation of consumption [8]. Some data might be obtained by postmortem examinations and reports. However, the number of cases and deaths involving NSO is likely underestimated due to the limited availability of updated and validated methods capable of detecting them. The interpretation is further hampered by the low concentrations in biological samples and by the co-consumption of other drugs [9]. According to these limitations, the following information was extracted.

### 2.1 Experimental Studies in Human

No experimental studies on the human psychomotor performances after consumption of ACRYLF, OCF and FUF were available, although it is expected that their effect is similar to other narcotic-type analgesics [10]. Only experimental studies involving the administration of FENT, either transdermal or intravenous, were retrieved and are shown in Table **1**.

Two studies tested psychomotor performances using driving simulators or driving tests. The study of Menefee *et al.*, by a driving simulator, demonstrated no difference before and after the administration of slowly increasing doses of FENT (over a period of 4 weeks). However, the study was conducted on patients who were administered a chronic opioid treatment for non-cancer pain, with doses of up to 15 mg of oxycodone [11]. Also, mental flexibility, memory recall and attentiveness were shown by testing patients with psychomotor performances [11]. Similar results were obtained when testing patients enrolled in long-term non-cancer pain treatment: a non-inferiority was demonstrated with respect to controls, once those patients taking additional unreported drugs were excluded. No significant effect was seen on attention, reaction, visual orientation, motor co-ordination and vigilance [3, 12]. This is expected, given the well-described mechanisms of tolerance in opioids and FENT users [13, 14]. As shown for prescription opioids, it is likely that the recreational use of NSO, alternating high doses and abstinence periods, might result in a lower tolerance development and in a higher risk for driving [2].

Another driving simulator performance study only involved FENT in co-administration with ketamine. Due to this co-consumption and to the difficulties in relating results with other studies, this was not included in our Table [15].

A tracometer (steering task) was used on healthy volunteers administered 100 µg of intravenous FENT, and showed an impact of FENT, especially on the correct reaction time, *i.e*. the length of time to make a cognitive decision of which way to move the target. Motor impairment was seen until 120 minutes, with heavier effects than after the administration of diazepam [16]. With the same concentration and administration route, another study showed that the eye-hand co-ordination test was hampered 15 minutes after the administration of 0-100 µg/70 kg of FENT, though the eye-hand co-ordination returned to normal levels after 60 minutes [17].

An association between plasma levels and psychomotor performances was shown by Veselis *et al.*, who demonstrated impairment in all the tested psychomotor performances starting from a plasma concentration of 2.5 ng/mL, with effects on memory and visual processing even at lower concentrations [18]. On the contrary, the previous work of Ghoneim *et al*., with the administration of FENT at 200 µg, found little effect on memory, only with the Backward Digit Span and Tapping Board task [19]. However, in this study, the administration took place at weekly intervals, likely giving time for the central nervous system (CNS) to adapt to the administration of the drug.

In volunteers, FENT produced significant impairing effects on auditory reaction time, signal detection, sustained attention and some memory task performances even at doses of 0.2 µg/kg (thus, approximately 14 µg in 70-kg males). Despite the very low concentrations measured, around 1.91 ng/mL, these effects on psychomotor performances were seen in the absence of marked sedation [20].

Overall, the data obtained from the experimental studies seems to point to a severe impairing effect when FENT is consumed by naïve users, while lower risk is expected in the context of a therapeutical administration. The effect of sex/gender was rarely evaluated in the revised articles. The study of Jamison *et al.*, [21] found no relationship between gender and outcome of the neuropsychological tests, although the evaluation was performed on patients and not on healthy volunteers. Although demographic data was available in some studies [11, 12] the effect of sex/gender was not assessed.

Ocfentanyl was selected for a clinical evaluation for its anesthetic effects and studies conducted on humans showed a potency 2.5 times higher than FENT and 200 times higher than morphine, with analgesic effects and sedation of 3 µg/kg of OCF comparable to that of 5 µg/kg of FENT [22]. Analgesic effect and respiratory depression peaked at 6 minutes and lasted approximately 1 hour [22], though this was not confirmed by [23].

Effects were dose-related, with a loss of consciousness described at around 2 µg/kg [22]. Moreover, it showed a lower tendency to accumulate in body tissues and fluids and a separation between hypnotic and analgesic ED_50_ values [24]. These depressant effects on the CNS functions suggest a likely impairing effect on psychomotor performances, despite the lack of experimental data.

### 2.2 Driving Under the Influence (DUID) of Opioids Cases

Strong opioids, including FENT, tested positive in 17.3% of fatal road crashes in Australia, but the relative contribution of FENT and the concentration of the detected substance were unknown [25]. In the Recommendations for Toxicological investigation of DUID fatalities, due to an increased prevalence of FENT registered by different laboratories, FENT was included in the mandatory substances to test, with a confirmation cut-off in the blood of 0.5 ng/mL [26]. Fentanyl was the most commonly detected drug (around 40%) of pedestrian/bicycle traumas in 2017-2019 [27] and in the USA FENT positive-DUID cases rose from 1% in 2014 to 5% in 2018, being 3% in 2019 [28]. An increase was also reported most recently, by the NMS Lab, that, by reviewing DUID cases over 11 years, revealed that 4.4% were positive for FENT, with a rise from 0.6% in 2010 to 12% in 2020 [29].

Considering also cases in which other drugs were detected, concentrations of FENT in these studies ranged from 0.1 to 157 ng/mL [28], until a maximum of 310 ng/mL [29].

Even though it was reported that the crash risk is not high for opioids, FENT included [30], among 20 cases of impaired driving with FENT-only intake reported by Rohrig *et al.*, 55% of drivers were found unresponsive in their vehicle, 55% left the roadway or lane of travel or showed erratic driving with unsteadiness, un-balance, impairment in walk and turn or one leg stand test, lethargy, and 8% involved a crash. Fentanyl median concentration was 3.7 ng/mL, ranging from 2.0 ng/mL to 16 ng/mL, thus 2 ng/mL was suggested as a starting level of impairment [28]. In the retrospective analysis performed by Hosokawa and Bierly, median FENT concentration tripled from 2010 (1.9 ng/mL) to 2020 (5.3 ng/mL) and the observations performed by the Drug Recognition Experts included poor balance (87%), poor co-ordination (80%), flaccid muscle tone (73%) slow speech and droopy eyelids (67% and 60%, respectively) [29].

It is worthwhile of consideration that in many cases of a road crash, FENT could be administered in the hospital after the road injuries [31], thus making challenging the evaluation of the prevalence of the substance within DUID cases as well as the estimation of its impact on psychomotor performances.

To the best of our knowledge, no real DUID case involving ACRYLF, OCF or FUF was described, although other fentanyl analogues, *e.g*. acetylfentanyl or butyrylfentanyl, were sometimes co-administered with FENT [29].

### 2.3 Intoxication Cases

Intoxications are another means to understand the effects of substances on those psychomotor performances which are important for driving. Several intoxications and fatal cases connected to FENT have been reported in the literature [32], with sudden collapse after inhalation of patch [33] or evidence of drowsy or altered mental state until coma. Motor weakness with as low as two patches was also reported among intoxication cases [34]. Since more abundant literature for FENT was found regarding experimental studies and cases of impaired driving, the present subsection will mainly focus on the other FENT-related molecules. Cases of intoxication by ACRYLF, OCF or FUF are reported in Table **2**.

Twenty-one intoxications associated with ACRYLF were reported by Sweden to the EMCDDA, even though no analytical confirmation was available [35]. This means that patients might have ingested other substances in addition to the analyte of interest. Reported symptoms included unconsciousness in 10 out 19 cases, while restlessness/anxiety was reported in 3 cases. Blurred vision was described only in 1 case, together with hallucinations, tiredness and muscular symptoms, but the victim also likely consumed stimulants.

This pattern of effects, mainly leading to various grades of CNS depression was confirmed in 8 intoxications (9 considering one involving also chloroisobutyrfentanyl) reported by the STRIDA project [36] and by a number of deaths, in which signs of respiratory depression were noted [37-39]. In both casuistries, ACRYLF was mainly consumed as nasal spray and, according to online and Intern forums, sub-milligram doses are enough to have psychoactive effects by this route [35, 36].

Particularly, case #1 had dizziness, paresthesia and tremor but no alteration of the reaction level scale (RLS; [42]), while cases #2, #5, #6, #7 and #8 had a certain grade of CNS depression until unconsciousness even with lower serum levels and similar or even higher sampling time. Particularly, case #8 was the only one reporting a female intoxication, showing an unknown grading of CNS depressant effect. Although sampling time and levels in serum were similar to case #1, who was alert, urinary levels were different and the influence of sex/gender cannot be estimated on the basis of a single case.

FUF was involved in one acute intoxication reported by the STRIDA Project [40], with very high concentrations compared to ACRYLF and with no psychomotor impairment observed (case #9 in Table **2**). No sedation was reported by a user in a forum around 250 µg, who also described strong nausea. Other users reported that FUF “worked” with a sedative effect of around 1-1.2 mg administered by nasal spray. Many users described a short duration of action followed by longer sedation, lasting around 1 hour and a half [43].

Among adverse effects notable for psychomotor performances, paranoia, psychosis and agitation have been reported after the use of OCF, even if this might be due to co-consumed drugs [22, 44]. Users reported a quick onset, in about 3 minutes, a “stimulant” effect and the early appearance of withdrawal symptoms [45]. Even though cases of death due to OCF were reported [9, 22, 32, 46-48], deaths were non-witnessed and cannot provide a picture of the symptoms. The low number of non-fatal intoxications does not speak in favor of low toxicity of the compound, and, given the described sudden loss of consciousness reported after snorting OCF [41], the possibility of fatal collapses while driving has to be considered.

This experimental section is aimed to evaluate the sensorimotor effects of new Fentanyl derivatives (ACRYLF, OCF and FUF) in the mouse model using behavioural tests of the “safety pharmacology protocol”, widely used to characterise new molecules. The results of these tests could validate this experimental protocol to predict the effects of opioids on human visual-motor and auditorial functions and their impact on human daily tasks with a particular focus on driving.

## 3 MATERIALS AND METHODS

### 3.1 Animals

Male ICR (CD-1^®^) mice weighing 30-35 g (Centralized Preclinical Research Laboratory, University of Ferrara, Italy) were group housed (5 mice per cage; floor area per animal was 80 cm^2^; minimum enclosure height was 12 cm), exposed to a 12:12-h light-dark cycle (light period from 6:30 AM to 6:30 PM) at a temperature of 20-22°C and humidity of 45-55% and were provided ad libitum access to food (Diet 4RF25 GLP; Mucedola, Settimo Milanese, Milan, Italy) and water. The experimental protocols performed in the present study were in accordance with the U.K. Animals (Scientific Procedures) Act of 1986 and associated guidelines and the new European Communities Council Directive of September 2010 (2010/63/EU), a revision of Directive 86/609/EEC. Experimental protocols were approved by the Italian Ministry of Health (license n. 335/2016-PR) and by the Animal Welfare Body of the University of Ferrara. According to the ARRIVE guidelines, all possible efforts were made to minimise the number of animals used, minimise the animals’ pain and discomfort and reduce the number of experimental subjects.

### 3.2 Drug Preparation and Dose Selection

FENT, ACRYLF, OCF and FUF were purchased from LGC Standards (Sesto San Giovanni, Milan, Italy). Naloxone (NLX) was purchased from Tocris (Bristol, UK). All the compounds were dissolved in a saline solution (0.9% NaCl) that was also used as the vehicle. Drugs were administered by intraperitoneal (i.p.) injection at a volume of 4 ul/g. The opioid receptor antagonist NLX (6 mg/kg, i.p.) was administered 15 mins before FENT, ACRYLF, FUF and OCF injections. The range of doses of FENS tested (0.01-15 mg/kg i.p.) was chosen based on our previous study [49].

### 3.3 Sensorimotor Tests

The effects of the three FENS were investigated using a battery of behavioural tests widely used in pharmacology safety studies for the preclinical characterization of new psychoactive substances in rodents [50-55]. All experiments were performed between 8:30 AM and 2:00 PM. Experiments were conducted blindly by trained observers working in pairs [55]. Mouse behavior was videotaped and analysed offline by a different trained operator who gives test scores.

We studied the voluntary and involuntary sensorimotor responses of the mice resulting from different reactions to visual, acoustic and tactile stimuli [51].

#### 3.3.1 Evaluation of the Visual Response

The visual response was verified by two behavioural tests that evaluated the ability of the mice to capture visual information when they are moving (the visual placing response) or when they are stationary (the visual object response). The visual placing response test is performed using a tail suspension modified apparatus able to bring the mouse toward the floor at a constant speed of 10 cm/s [51]. The downward movement of the mouse is videotaped by a camera. A frame-by-frame analysis allows one to evaluate the beginning of a mouse’s reaction while it is close to the floor. When the mouse starts to react, an electronic ruler evaluates the perpendicular distance in millimetres from the eyes of the mouse to the floor. The untreated control mouse perceives the floor and prepares to come into contact with it at a distance of 27 ± 4.5 mm. The visual placing response was measured at 0, 15, 35, 70, 125, 185, 245 and 305 min post-injection. A visual object response test was used to evaluate the ability of the mouse to see an object approaching from the front or the side, thus inducing the animal to shift or turn its head or retreat it [51]. For the frontal visual response, a white horizontal bar was moved in front of the mouse’s head; the manoeuvre was repeated three times. For the lateral visual response, a small dentist’s mirror was moved into the mouse’s field of view in a horizontal arc until the stimulus was between the mouse’s eyes. The procedure was conducted bilaterally and was repeated three times. A score of 1 was assigned if there was a reflection in the mouse movement; otherwise, a score of 0 was assigned. The total value was calculated by adding the scores obtained for the frontal and lateral visual object responses (overall score 9). The visual object response was measured at 0, 10, 30, 60, 120, 180, 240 and 300 min post-injection.

#### 3.3.2 Evaluation of the Acoustic Response

Acoustic response measures the reflex of the mouse in response to an acoustic stimulus produced behind the animal. In particular, four acoustic stimuli of different intensities and frequencies were tested [51]. Each sound test was repeated three times. A score of 1 was given if there was a response and a score of 0 was given if there was no response, for a total score of 3 for each sound. The total acoustic score was calculated by adding scores obtained in the four tests (overall score 12). The acoustic response was measured at 0, 10, 30, 60, 120, 180, 240 and 300 min post injection.

#### 3.3.3 Evaluation of the Tactile Response

The tactile response of each mouse was verified through vibrissae, pinna and corneal reflex, as previously described [51]. Data is expressed as the sum of the three parameters mentioned above. The vibrissae reflex was evaluated by touching the vibrissae (right and left) with a thin hypodermic needle once per side. A score of 1 was given if there was a response (turning the head to the side of the touch) or a score of 0 was given if there was no response (overall score 2). The pinna reflex was assessed by touching the pavilions (left and right) with a thin hypodermic needle. First the interior pavilions and then the external pavilions were stimulated. This test was repeated twice per side. A score of 1 was given if there was a response and a score of 0 was given if there was no response (overall score 4). The corneal reflex was assessed by gently touching the cornea of the mouse bilaterally with a thin hypodermic needle and evaluating the response. A score of 1 was given if the mouse moved only its head, 2 if it only closed the eyelid and 3 if it both closed the eyelid and moved the head (overall score 6). Each tactile response was measured at 0, 10, 30, 60, 120, 180, 240 and 300 min post-injection.

### 3.4 Data and Statistical Analysis

Data are expressed in arbitrary units (visual objects response; acoustic response; vibrissae, corneal and pinna reflex) or percentage of baseline (visual placing response). The statistical analysis of the effects of the individual substances in different concentrations over time and that of antagonism studies were performed using a two-way ANOVA followed by a Bonferroni test for multiple comparisons. The statistical analysis was performed using Prism software (GraphPad Prism, USA). All analyses were performed using GraphPad Prism software.

Dose-response curves were used to calculate the ED_50_ in each test, using Prism software (GraphPad Prism, USA)

## 4 RESULTS

### 4.1 Evaluation of the Visual Object Response

**Fig. (2):**
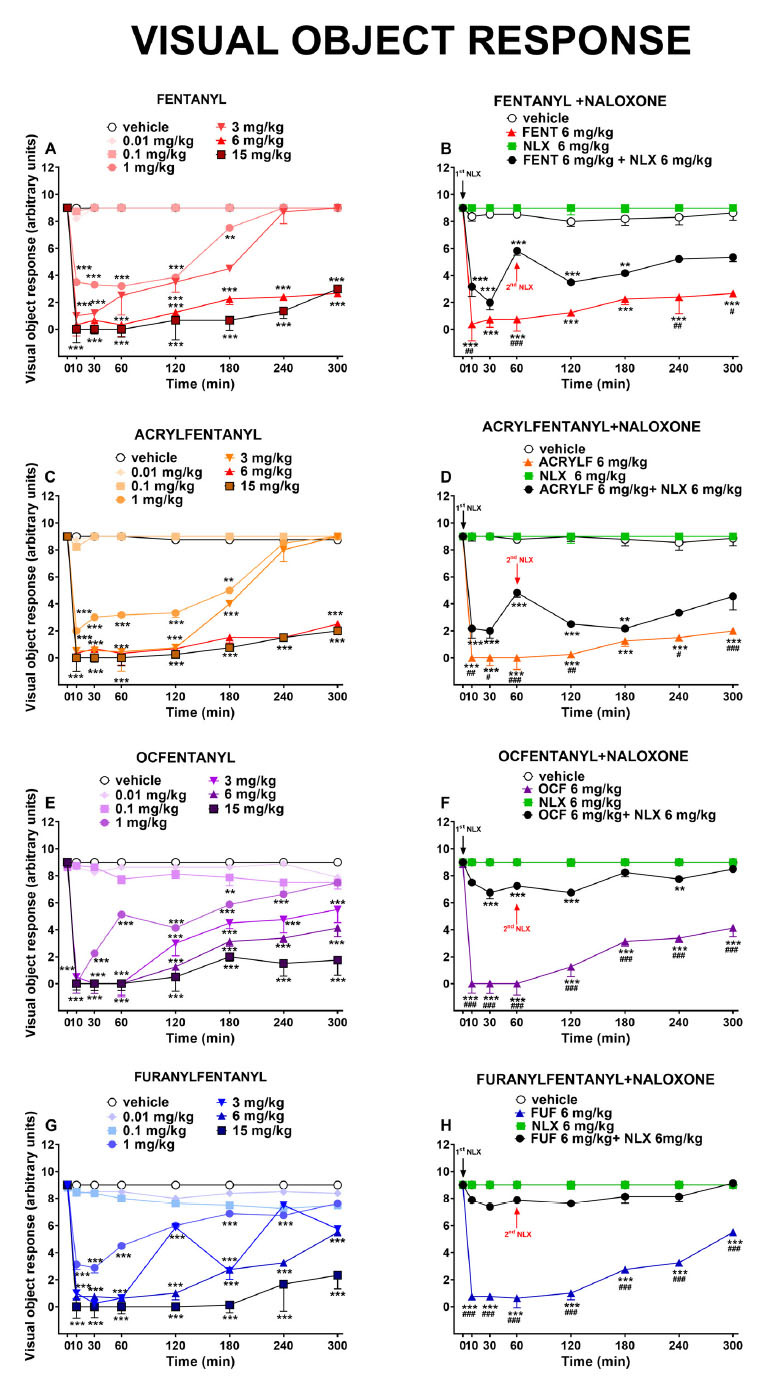
Effect of acute systemic administration (0.01-15 mg/kg i.p.) of FENT (panel **A**); ACRYLF (panel **C**); OCF (panel **E**) and FUF (panel **G**) on the visual object response test of the mouse. Interaction of effective dose of all the compounds (6 mg/kg) with the opioid receptor antagonist NLX (6 mg/kg, i.p.; respectively panels **B-D-F-H**). Data are expressed as arbitrary units (see materials and methods) and represent the mean ± SEM of 8 determinations for each treatment. Statistical analysis was performed by two-way ANOVA followed by Bonferroni’s test for multiple comparisons. *p<0.05, **p<0.01, ***p<0.001 *versus* vehicle; #*p<*0.05, ##*p<*0.01, ###*p****<***0.001 *versus* NLX+ agonist.

The visual object response was not affected in mice treated with the vehicle (Fig. **2**).

Acute systemic administration of the four opioids (0.01-15 mg/kg i.p.) dose-dependently decreased the visual object responses of mice. After the administration of FENT, ACRYLF, OCF and FUF the visual object response was significantly affected by treatment: FENT [F_6,392_ = 359.4, *P*<0.0001], time [F_7,392_ = 84.90, *P*<0.0001] and time × treatment interaction [F_42,392_ = 14.98, *P*<0.0001]; ACRYLF [F_6,392_ = 502.4, *P*<0.0001], time [F_7,392_ = 122.9, *P*<0.0001] and time × treatment interaction [F_42,392_ = 22,13, *P*<0.0001]; OCF [F_6,392_ = 324.5, *P*<0,0001], time [F_7,392_ = 84.91, *P*<0.0001] and time × treatment interaction [F_42,392_ = 13.39, *P*<0.0001]; FUF [F_6,392_ = 418.5, *P*<0.0001], time [F_7,392_ = 108.3, *P*<0.0001] and time × treatment interaction [F_42,392_ = 18.58, *P*<0.0001]; (Fig. **2 A-C-E-G**).

The pre-treatment with NLX (6 mg/kg) totally prevented the inhibitory effects of FUF [F_1,112_ = 1407, *P*<0.0001], time [F_7,112_ = 81,97, *P*<0.0001] and time × treatment interaction [F_7,112_ = 38.83, *P*<0.0001] and partially for the other compounds. The injection of a second dose of NLX (6 mg/kg i.p.) did not totally prevent the visual alterations induced by FENT, ACRYLF and OCF: FENT [F_1,112_ = 63.71, *P*<0,0001], time [F_7,112_ = 32.44, *P*<0.0001] and time × treatment interaction [F_7,112_ = 3.064, *P*=0.0055]; ACRYLF [F_1,112_ = 84.89, *P*<0.0001], time [F_7,112_ = 68.27, *P*<0,0001] and time × treatment interaction [F_7,112_ = 4.786, *P*<0.0001]; OCF [F_1,112_ = 508.8, *P*<0.0001], time [F_7,112_ = 34.51, *P*<0.0001] and time × treatment interaction [F_7,112_ = 13.53, *P*<0.0001]; (Fig. **2 B-D-F-H**). The comparison of the dose-response curves of all compounds in the sensorimotor tests performed in this study are represented in Fig. **6 (A-B-C-D)**. In particular, the comparison of the dose-response curves (Fig. **6A**) of all compounds in the visual object response test revealed the following rank of potency: OCF (ED_50_= 1.60 mg/kg) ≥ ACRYLF (ED_50_= 1.97 mg/kg) ≥ FUF (ED_50_= 2.17 mg/kg) = FENT (ED_50_= 2.7 mg/kg).

### 4.2 Evaluation of the Visual Placing Response

**Fig. (3):**
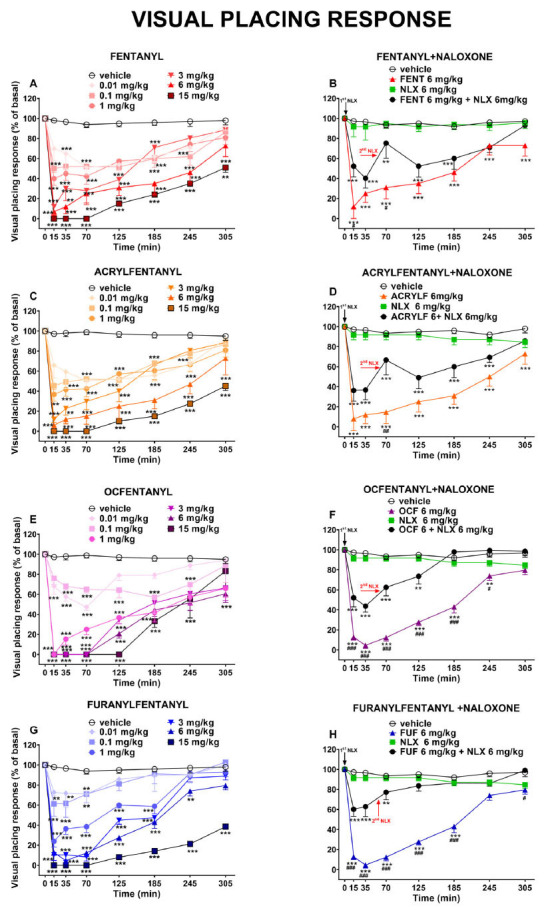
Effect of acute systemic administration (0.01-15 mg/kg i.p.) of FENT (panel **A**); ACRYLF (panel **C**); OCF (panel **E**) and FUF (panel **G**) on the visual placing response test of the mouse. Interaction of effective dose of all compounds (6 mg/kg) with the opioid receptor antagonist NLX (6 mg/kg, i.p.; respectively panels **B-D-F-H**). Data are expressed as a percentage of baseline (see material and methods) and represent the mean ± SEM of 8 determinations for each treatment. Statistical analysis was performed by two-way ANOVA followed by the Bonferroni’s test for multiple comparisons. *p<0.05, **p<0.01, ***p<0.001 *versus* vehicle; #*p<*0.05, ##*p<*0.01, ###*p****<***0.001 *versus* NLX + agonist.

The visual placing response was not affected in mice treated with the vehicle (Fig. **3**).

Acute systemic administration of the four opioids (0.01-15 mg/kg i.p.) dose-dependently decreased the visual placing responses of mice. After the administration of FENT, ACRYLF, OCF and FUF the visual placing response was significantly affected by treatment: FENT [F_6,392_ = 85.91, *P<*0.0001], time [F_7,392_ = 73.13, *P<*0.0001] and time × treatment interaction [F _42,392_ = 4.023, *P<*0.0001]; ACRYLF [F_6,392_ = 91.91, *P<*0.0001], time [F_7,392_ = 73,86, *P<*0,0001] and time × treatment interaction [F_42,392_ = 4.079, *P<*0.0001]; OCF [F_6,392_ = 115.3, *P<*0.0001], time [F_7,392_ = 94.78, *P<*0,0001] and time × treatment interaction [F_42,392_ = 6.645, *P<*0.0001]; FUF [F_6,388_ = 184.5, *P<*0.0001], time [F_7,388_ = 123.1, *P<*0.0001] and time × treatment interaction [F_42,388_ = 8.587, *P<*0.0001]; (Fig. **3A-C-E-G**).

The pre-treatment with NLX (6 mg/kg) partially prevented the inhibitory effect of all the compounds. The injection of a second dose of NLX (6 mg/kg i.p.) did not totally prevent the visual alteration induced by the agonists however it reduced it slightly in the last hours of measurements: FENT [F_3,224_ = 66.85, *P<*0.0001], time [F_7,224_ = 11.50, *P<*0.0001] and time × treatment interaction [F_21, 224_ = 3.636, *P<*0.0001]; ACRYLF [F_3,224_ = 106.0,.*P<*0.0001], time [F_7,224_ = 15.31, *P<*0.0001] and time × treatment interaction [F_21,224_ = 5.137, *P<*0,0001]; OCF [F_3,224_ = 212.6, *P<*0,0001], time [F_7,224_ = 42.81, *P<*0.0001] and time × treatment interaction [F_21,224_ = 16.56, *P<*0.0001] FUF [F_3,224_ = 277.6, *P<*0.0001], time [F_7,224_ = 41.65, *P<*0.0001] and time × treatment interaction [F_21,224_ = 18.88, *P<*0,0001]; (Fig. **3 B-D-F-H**)].

The comparison of the dose-response curves (Fig. **6B**) of all compounds in the visual placing response test revealed the following rank of potency: OCF (ED_50_= 0.88 mg/kg) > ACRYLF (ED_50_= 1.38 mg/kg) ≥ FENT (ED_50_= 1.87 mg/kg) > FUF (ED_50_= 2.51 mg/kg).

### 4.3 Evaluation of the Acoustic Response

**Fig. (4):**
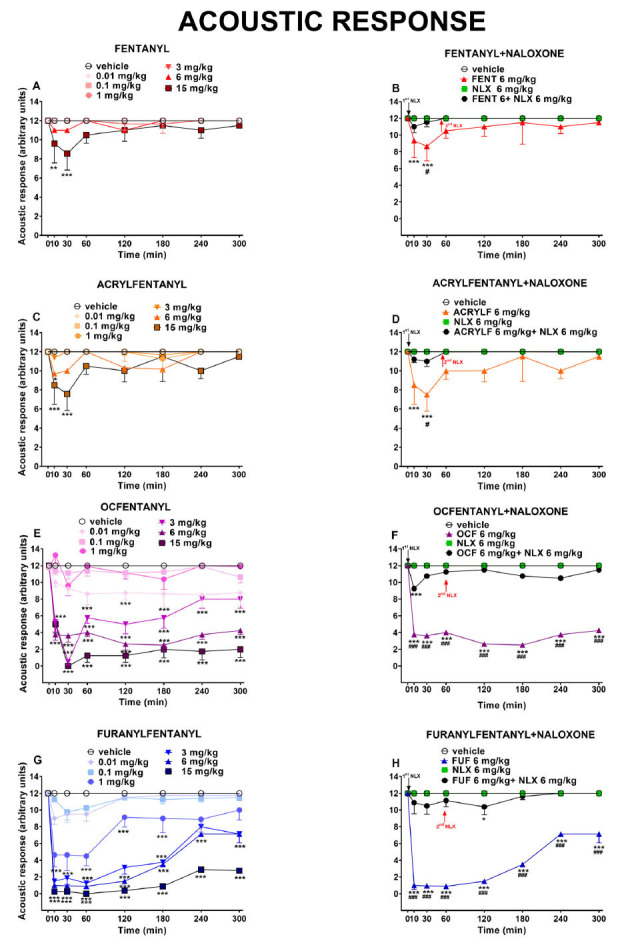
Effect of acute systemic administration (0.01-15 mg/kg i.p.) of FENT (panel **A**); ACRYLF (panel **C**); OCF (panel **E**) and FUF (panel **G**) on the acoustic response test of the mouse. Interaction of effective dose of all compounds (6 mg/kg) with the opioid receptor antagonist NLX (6 mg/kg, i.p.; respectively panels **B-D-F-H**). Data are expressed as arbitrary units (see material and methods) and represent the mean ± SEM of 8 determinations for each treatment. Statistical analysis was performed by two-way ANOVA followed by the Bonferroni’s test for multiple comparisons for both the dose response curve of each compounds at different times. *p<0.05, **p<0.01, ***p<0.001 *versus* vehicle; #*p<*0.05, ###*p<*0.001 *versus* NLX+ agonist.

The acoustic responses did not change in vehicle-treated mice over the 5-h observation (Fig. **4**). Acute systemic administration of FENT and its derivatives (0.01-15 mg/kg) decreased the acoustic responses in mice. In particular, the administration of FENT and ACRYLF decreased the acoustic response only at the highest dose tested and the effect disappeared after 60 min of treatments at the highest dose tested; with OCF and FUF the acoustic response was significantly affected by treatment: FENT [F_6,392_ = 7.932, *P<*0.0001], time [F_7,392_ = 1.442, *P=*0.18^71^] and time × treatment interaction [F_42,392_ = 0.8902, *P=*0.66^85^]; ACRYLF [F_6,392_ = 11.08, P < 0.0001], time [F_7,392_ = 2.461, P = 0.0176] and time × treatment interaction [F_42,392_ = 1.289, P = 0.1144]. In difference to FENT and ACRYLF, OCF and FUF induced a dose-dependent inhibition of the acoustic reflexes and the effect persisted up to 5 hours of measurements: OCF [F_6,392_ = 177.0, *P<*0.0001], time [F_7,392_ = 29.51, *P<*0.0001] and time × treatment interaction [F_42,392_ = 5.545, *P<*0.0001]; FUF [F_6,392_ = 264.8, *P<*0.0001], time [F_7,392_ = 79.92, *P<*0.0001] and time × treatment interaction [F_42,392_ = 8.625, *P<*0.0001]; (Fig. **4 A-C-E-G**).

The pre-treatment with NLX (6 mg/kg) prevented the inhibitory effects of FENT, ACRYLF and FUF while partially with OCF. The injection of a second dose of NLX (6 mg/kg i.p.) reverted the effect of OCF: FENT [F_3,224_ = 5.802, *P=*0.000^8^], time [F_7,224_ = 0.9575, *P=*0,46^32^] and time × treatment interaction [F_21,224_ = 0.5618, *P=*0.9402]; ACRYLF [F_3,224_ = 12.22, *P<*0.0001], time [F_7,224_ = 1.716, *P=*0.10^63^] and time × treatment interaction [F_21,224_ = 1.027, *P=*0.4315] and OCF [F_3,224_ = 461.2, *P<*0.0001], time [F_7,224_ = 14.23, *P<*0.0001] and time × treatment interaction [F_21,224_ = 10.47, *P<*0.0001]; FUF [F_3,224_ = 628.8, *P<*0,0001], time [F_7,224_ = 30.34, *P<*0.0001] and time × treatment interaction [F_21,224_ = 21.74, *P<*0.0001]; (Fig. **4B**-**D**-**F**-**H**)].

The comparison of the dose-response curves (Fig. **6C**) between OCF and FUF revealed a major potency for FUF (ED_50_= 2.40 mg/kg) in comparison to OCF (ED_50_= 4.36 mg/kg). The ED_50_ was not determined for the rest of the compounds.

### 4.4 Evaluation of the Tactile Response

**Fig. (5):**
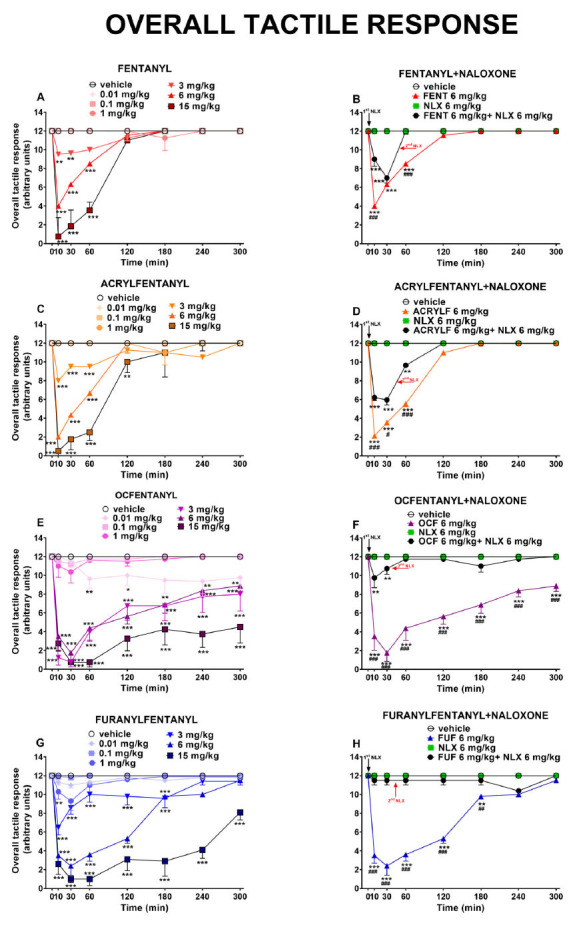
Effect of acute systemic administration (0.01-15 mg/kg i.p.) of FENT (panel **A**); ACRYLF (panel **C**); OCF (panel **E**) and FUF (panel **G**) on the overall tactile response. Interaction of effective dose of all compounds (6 mg/kg) with the opioid receptor antagonist NLX (6 mg/kg, i.p.; respectively panels **B-D-F-H**). Data are expressed as arbitrary units (see material and methods) and represent the mean ± SEM of 8 determinations for each treatment. Statistical analysis was performed by two-way ANOVA followed by Bonferroni’s test for multiple comparisons. *p<0.05, **p<0.01, ***p<0.001 *versus* vehicle; #*p<*0.05, ##*p<*0.01, ###*p****<***0.001 *versus* NLX+ agonist.

The overall tactile responses (pinna, vibrissae, cornea) did not change in vehicle-treated mice over the 5-h observation (Fig. **5**). Acute systemic administration of FENT and its derivatives (0.01-15 mg/kg) decreased in a dose-dependent manner the overall tactile responses in mice. In particular, after the administration of FENT, ACRYLF, OCF and FUF the tactile response was significantly affected by treatment FENT [F_6,392_ = 82.00, *P<*0.0001], time [F_7,392_ = 56.06, *P<*0.0001] and time × treatment interaction [F_42,392_ = 19.30, *P<*0.0001]; ACRYLF [F_6,392_ = 88.29, *P<*0.0001], time [F_7,392_ = 57.88, *P<*0.0001] and time × treatment interaction [F_42,392_ = 18.74, *P<*0.0001]; OCF [F_6,392_ = 116.5, *P<*0.0001], time [F_7,392_ = 22.42, *P<*0.0001] and time × treatment interaction [F_42,392_ = 4.863, *P<*0.0001]; FUF [F_6,392_ = 231.4, *P<*0.0001], time [F_7,392_ = 48.47, *P<*0,0001] and time × treatment interaction [F_42,392_ = 11.52, *P<*0.0001]; (Fig. **5 A-C-E-G**).

The pre-treatment with NLX (6 mg/kg) totally prevented the inhibitory effects induced by FUF [F_3,224_ = 324.6, *P<*0.0001], time [F_7,224_ = 31,34, *P<*0,0001] and time × treatment interaction [F_21,224_ = 30.30, *P<*0.0001] and partially with the other compounds. The injection of a second dose of NLX (6 mg/kg i.p.) reverted totally the tactile responses inhibition induced by the three agonists: FENT [F_3,224_ = 251.6, *P<*0.0001], time [F_7,224_ = 169.9, *P<*0,0001] and time × treatment interaction [F_21,224_ = 74.62, *P<*0.0001]; ACRYLF [F_3,224_ = 735.8, *P<*0.0001], time [F_7,224_ = 451.3, *P<*0.0001] and time × treatment interaction [F_21,224_ = 171.7, *P<*0,0001]; OCF [F_3,224_ = 209.2, *P<*0.0001], time [F_7,224_ = 13.50, *P<*0,0001] and time × treatment interaction [F_21,224_ = 9.328, *P<*0.0001]; (Fig. **5 B-D-F-H**).

**Fig. (6):**
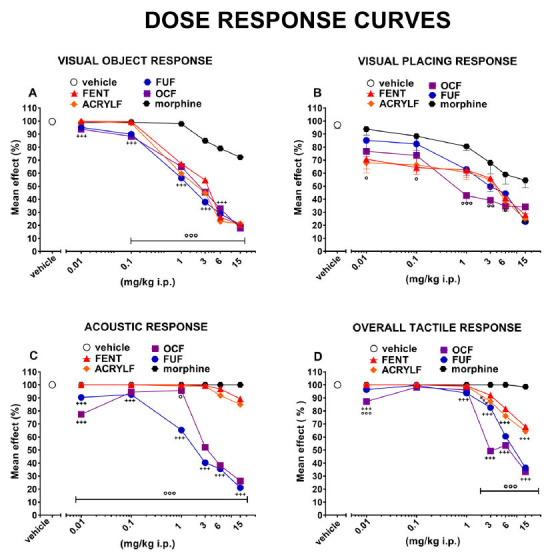
Dose-response curves of FENT, ACRYLF, OCF and FUF on the visual object response (panel **A**); the visual placing response (panel **B**); the acoustic response (panel **C**); the tactile response (panel **D**). Data of morphine were elaborated in [59]. Data are expressed in percent (%) and represent the mean ± SEM of 8 determinations for each treatment. Statistical analysis was performed by two-way ANOVA followed by Bonferroni’s test for multiple comparisons. **+++***p<*0.001 *versus* FENT; °*p<*0.05, °°*p<*0.01, °°°*p<*0.001 *versus* morphine.

The comparison of the dose-response curves (Fig. **6D**) between OCF and FUF in the tactile response test revealed a major potency for OCF (ED_50_= 5.85 mg/kg) in comparison to FUF (ED_50_= 9.20 mg/kg). The ED_50_ was not determined for the rest of the compounds.

## 5 DISCUSSION

### 5.1 Visual Object and Visual Placing Responses

The results of the acute systemic administration of FENT, ACRYLF, OCF and FUF on the startle response to visual stimuli demonstrate that opioid receptors, in particular mu receptors, play an important role in modulating the visual responses of mice after opioid injections [57-59]. Indeed, we have demonstrated in our previous study that the inhibitory effects of morphine and its analogue (MT-45) on the visual object and placing tests were totally prevented by the pre-treatment with NLX [59]. In this case, the pre-treatment with NLX partially prevented the inhibitory effects induced by all the opioids in visual objects and visual placing tests. The administration of the second dose of NLX (6 mg/kg at 55 min after treatment) was not effective at blocking the inhibitory effects on visual reflexes induced by FENT, ACRYLF, OCF and FUF. The effect of FENS on the visual placing seemed to be more profound than those in the object test. In contrast to the latter, the visual placing test links the movement of the mouse to its visual perception. In particular, to perform the visual placing test the mouse needs to integrate the visual and tactile stimulus with the vestibular information to correctly extend the muscles of the neck and forelegs to land on the ground [60]. Moreover, studies in freely moving mice [61] and also rats [62] have found that eye movement patterns in these animals are often non-conjugate and these movements were systematically coupled to changes in orientation of the animal’s head with respect to the horizontal plane (head tilt). Eye movements in response to static tilt changes are associated with the otolith organs, which sense head acceleration, including gravity, and are referred to as “ocular countertilt” reflexes [63]. It is suggested by Meyer and coworkers, that these eyehead coupling movements in rodents could serve to stabilize the visual field with respect to the ground [64]. Thus, the involvement of the vestibular inputs and the spinal motoneurons in controlling posture and body movement in the face of gravity has been established [65, 66]. Eye movements in freely moving mice constantly stabilize the animal’s visual field by counteracting head rotations through the vestibulo-ocular reflex (VOR; [64, 67]), maintaining the large panoramic overhead view [63]. In mice administered fentalogs, this mechanism appears to be hampered and that could reveal the role of fentanyl in impairing the vestibule-ocular reflex.

Other studies on the mechanism of action of opioids in the medial vestibular nucleus have proved that opioids can induce direct excitatory actions after GABAergic inhibition [68] and this could explain the results of our study. Mu-opioid receptors have been detected in the retina of rodents [69, 70]. The acute systemic administration of opioids induced pupil constriction and reduced Pupillary Light Reflexes in mice. These effects were blocked by mu antagonist [71]. These findings also reveal that opioids can act directly with mu-opioid receptors of the retina and alter vision in mice [71, 72].

### 5.2 Acoustic Response

Our data shows that FENT, ACRYLF, OCF and FUF reduced acoustic response in mice in a dose-dependent manner. The effect of OCF and FUF was more potent and persistent compared to FENT and ACRYLF, and these differences could be related to their chemical structures [73, 74]. The pre-treatment with NLX prevented the acoustic alterations induced by all the opioids, revealing the involvement of mu-opioid receptors in the acoustic inhibitory effects induced by FENS [59]. It is important to highlight that in our previous study, morphine at the range dose of 0.01-15 mg/kg did not affect the acoustic reflexes of mice. However, the effect of FENS was robust and dose-dependent. The mechanism by which FENS could alter the acoustic responses is not yet elucidated. However, there is evidence in the literature demonstrating the expression of opioid peptides in the inner ear. In particular, mRNA for the mu-opioid receptor, delta-opioid receptor, and kappa-opioid receptor was detected in rat and guinea pig cochlea by RT‐PCR [75, 76]. In the mouse spiral ganglia neurons most of the neurons were immunoreactive to mu-opioid receptors. In the organ of Corti, mu-opioid receptors were expressed in inner hair cells (IHC) and outer hair cells (OHC), and the fibers underneath the IHC were also detected [77]. It has been reported that intoxication with opioids such as MT-45 and hydrocodone might cause gradual sensory deafness requiring a cochlear implant for the recovery of hearing [78, 79]. The temporary loss of hearing produced by methadone has also been reported [80, 81]. The fact that opioid receptors, in particular, mu ones are expressed in the inner ear of mice confirms their involvement in the acoustic alterations produced in mice after acute systemic administration of FENS and that could be related to temporary dysfunction of the cochlea, which was prevented by NLX pre-treatment in our test [82].

### 5.3 Tactile Response

Our data demonstrate that FENT, ACRYLF, OCF and FUF significantly reduced the tactile response of mice. Also, in this test, the effect of OCF and FUF is more potent and persistent in comparison to FENT and ACRYLF. Again these differences could be related to their chemical structures [49, 73, 74]. The pre-treatment with NLX did not totally block the inhibitory effects of FENT, ACRYLF and OCF. While the second dose of NLX totally blocked these effects. Our data revealed the role of mu-opioid receptors in inhibiting the tactile response in mice after opioid injections. Indeed, we demonstrated in our previous study that MT45 (synthetic substitute of morphine) but not morphine reduced the tactile response at the dose of 15 mg/kg and the effect was prevented by a pre-treatment with 6 mg/kg of NLX [59]. The tactile experience through the mystacial vibrissae (whiskers) is the main way to collect information from the outside environment for rodents. The mechanism by which opioids reduce the tactile response in mice is not yet defined. However, a recent elegant study demonstrated that FENT inhibits the Air Puff-Evoked sensory information of mice, acting *via* mu receptors on cerebellar neurons, by reducing GABAergic responses in molecular layer interneurons (MLIs) and Purkinje cells (PCs), through the cAMP-PKA signaling pathway [83]. These findings reveal the role of mu-opioid receptors distributed in the cerebellum area in controlling the sensory responses of rodents.

### 5.4 Translational Paradigm from Animal to Human in Cases of DUID

Our study is aimed to evaluate the effects of FENS on sensorimotor functions in mouse model and to validate our behavioral tests to predict the possible impact of FENS on human psychomotor performances, particularly in those involved in driving abilities.

Clearly, the inference of data obtained from animal models to humans presents several limitations and should be performed with caution. Keeping in mind the possible biases connected to this operation and the general difficulty in predicting the effects of drugs on the ability to drive on the basis of psychomotor tests, our study revealed substantial accordance between experimental and human data. Our experimental data reveals that FENS impairs sensorimotor functions in mice and that the effects were blocked by a repeated administration of a high dose of the mu-opioid receptor antagonist NLX. The visual placing test performed in mice allows to evaluate functions such as vision, co-ordination and proprioception, which are critical in the fitness and ability to drive. The response to visual stimuli and visual/motor tracking are at the basis of several psychomotor tests and driving measures, such as the simple braking reaction time, cue recognition reaction time, visual retention test and trail-making test, the impairment of which could be considered predictive of an inability to drive and was therefore tested by several authors [11]. FENT impaired eye-hand coordination in humans [17] and the Critical Flicker Fusion Task [18], which tests visual processing and visual-motor skills. Data retrieved from intoxication cases accordingly showed some impairing in the visual functions, with hallucinations and blurred visions reported in cases of ACRYLF intoxications [35]. It appears obvious that when the visual acuity is impaired, the proprioception and the motor response might not be adequate to the external stimuli.

Acoustic perception, as well as a visual one, has been shown to influence the driving speed and motion perception [84], which is in turn connected to braking responses and driving performances [85]. A reduction in in-car noises of 5dB has been shown to lead to a speed underestimation, with potential effect on the risk of crashing [84]. Hearing impairment is associated with road accidents [86], while auditory advisory information seems to be linked to quicker driver responses [87], suggesting that acoustic cues are fundamental for drivers. Touch is less stimulated, compared to vision during driving and, for this reason, it has been targeted as a sense through which is possible to vehicolate feedback to the driver [88]. However, tactile sensations are fundamental for proprioception, as in the berg balance test, in which the patient has to stand with an eye closed on one foot [11]. Considering experimental cases on humans, low doses of FENT impair auditory, reaction time, signal detection, sustained attention and some memory task performances even in the absence of marked sedation [20]. Accordingly, in our animals, some FENS impaired auditory and tactile responses of mice at low doses (0.01 and 0.1 mg/kg) that did not impair or facilitate spontaneous [89] and stimulated motor activity [49] of mice.

Moreover, our study demonstrated that a single dose of NLX did not block the effects of FENS on sensorimotor functions. While a second dose prevented most of these effects. This is in line with preclinical [49] and clinical reports that suggest repeated administration of NLX dosing in cases of FENT intoxications, in order to avoid the reappearance of its effects [90]. It is worth speculating that the human and the mouse mu-opioid receptors, share a high (94%) level of protein similarity [91]. Yet, a direct comparison of the pharmacological profile of a panel of mu-opioid ligands on the rat *vs*. human [92] and on the rhesus *vs*. human [93] mu opioid receptors, demonstrated very high levels of correlation. In addition, affinity values of a series of N-alkyl benzomorphans were obtained in cells expressing the mouse mu opioid receptor and compared to that of rat and monkey cortex showing again high levels of correlation [94]. Collectively, these findings prove the validity of the mouse model to predict the effects of opioids on humans, particularly, on sensorimotor impairments, and to improve the possible therapeutic interventions in the case of DUID.

## CONCLUSION

In the present work, the experimental study performed on a mouse model has shown sensorimotor alterations, including visual, acoustic and tactile responses, induced by fentanyl and by three fentalogs, acrylfentanyl, ocfentanyl and furanylfentanyl. The results are in accordance with the data that emerged from the revision of the literature regarding experimental data on humans, driving under the influence of drugs and intoxication cases, suggesting that novel synthetic opioids might affect the psychomotor performances involved in driving.

## Figures and Tables

**Table 1:** Experimental studies.

**Author**	**Substance**	**Study**	**Sample**	**Dose, Duration, Levels**	**Performance Tested**	**Results**
Ghoneim *et al.*, 1975 [19]	FENT iv	Placebo- and diazepam-controlled, pretest-posttest	10 healthy volunteers (M)	0.1-0.2 mg at weekly intervals	Backward Digit Span, Tapping Board, Serial Learning, Short-Term Memory, Delayed Recall, Simple Reaction Time, Choice Reaction Time, Visual Retention Test at 2, 6 and 8 h	0.2 mg of FENT affected Digit Span and Tapping board at 2h
Stevenson *et al.*, 1986 [16]	FENT iv	Placebo- and diazepam-controlled, double-blind, crossover design	9 healthy volunteers (5M, 4F)	0.1 mg	Tracometer (steering task) measuring reaction time, nonovershoot movement time, total response time, overshoot movement time, frequency of errors, frequency of overshoots	Effect of both drugs in all tests, with slower reaction times with FENT, compared to diazepam
Veselis *et al.*, 1994 [18]	FENT iv	Placebo-controlled, randomized, pretest-posttest	9 healthy volunteers (5M, 4F)	1, 1.5, 2.5 ng/mL	Memory by Rey Auditory-Verbal Recall Task (Rey AVLT), Picture Recall. Psychomotor by Critical Flicker Fusion Task (CFFT), Choice Reaction Time (CRT), Digit Symbol Substitution Test (DSST), Serial Numbers (SN)	Dose-dependent effects on memory. Below 2.5 ng/ml, only alteration of CFFT. Over 2.5 ng/ml, all performances were altered, with a decrement of 15-30%
Zacny *et al.*, 1992 [17]	FENT iv	Placebo-controlled, randomized, double-blind, crossover design	13 healthy volunteers (10 M, 3 F)	0-0.1 mg/70 kg	Maddox Wing (MW), auditory reaction time (ART), eye-hand co-ordination. Tests at 15 and 60 min post-injection	Altered eye-hand co-ordination 15 min post-injection. No other effect
Schneider *et al.*, 1999 [20]	FENT	Placebo-controlled	24 healthy volunteers (M)	0.2 μg/kg, with plasma level of 1.91 ± 1.17 ng/mL after 15 min and 0.67 ng/mL ± 0.23 after 30 min	Divided attention, reaction time measurement (Vienna Reaction Time), signal detection, sustained attention (Pauli test), memory (WIT)	Significant differences in reaction time in response to auditory input, signal detection hit, sustained attention and memory by using a distractor
Jamison *et al.*, 2003 [21]	FENT td	Prospective, oxycodone-controlled, pretest-posttest	144 patients with low back pain (39.6% F)	Average 42.6 μg±19.0 and 43.7 μg ± 21.7	DSST and Trail Making Test-B 90 and 180 days after administration	Improvement of psychomotor performances
Sabatowski *et al.*, 2003 [12]	FENT td	Prospective, case-control, randomized	30 patients with chronic non-cancer pain (18 M, 12 F) *vs.* 90 healthy subjects	Median 50 ug/hour, 44 days, 1.35 ng/mL	Attention test (COG), test for reaction time under pressure or determination test (DT), test for visual orientation (TAVT), test for motor co-ordination (2-Hand), vigilance test (VIG)	Non-inferiority with respect to control
Menefee *et al.*, 2004 [11]	FENT td	Prospective, single group pretest-posttest	23 patients (17 M, 6 F) on short-acting opioids (up to 15 mg oral oxycodone) for chronic non-cancer pain	Increase of 25 μg/h per week for 4 weeks (maximum dose 125 μg/h)	Driving task for simple braking reaction time, cue recognition reaction time, destination driving, and evasive action, visual motor tracking/mental flexibility by the Trail Making test A and B, memory by Rey Complex figure test and recognition trial and WMS-III. Attention by d2 Test of Attention and CPT-II. Balance by Berg Balance Test	No difference pre-post FENT for driving tasks. No decrease in performance, but improvement in mental flexibility, immediate and 20-minute memory recall, focus and attentiveness

**Abbreviations**: FENT: fentanyl. M: males; F: females. iv: intravenous; td: transdermal. h: hours; min: minutes. Wilde intelligence test (WIT) Weschler Memory Scale-III Spatial Span test (WMS-III) Conner’s Continuous Performance Test II [CPT-II].

**Table 2:** Acute non-fatal intoxications.

**Substance**	**Dose (Self-Reported)**	**Serum**	**Psychomotor Performaces**	**Case or ** **Sample**	**Sampling Time**	**Author**
ACRYLF	-	-	Tiredness, somnolence, unconsciousness, anxiety, hallucinations, blurred vision	N=21 (18 M, 3 F)	-	EMCDDA, 2017 [35]
ACRYLF	20 mg/day x 4 days	1.3 ng/mL	Dizziness, paresthesia, tremor	#1 M	2 h	Helander *et al.*, 2017 [36]
ACRYLF	1 spray	0.6	Drowsy or confused	#2 M	6.5	
ACRYLF	1 spray	ND	-	#3 M	1.5 h	
ACRYLF	-	1.0	Psychotic behavior (agitation and delirium)	#4 M	1.5 h	
ACRYLF	-	2.1	CNS depression	#5 M	-	
ACRYLF		0.7	GCS 3	#6 M	14 h	
ACRYLF	6-8 spray	0.8	Very drowsy or confused	#7 M	-	
ACRYLF	-	1.3	CNS depression	#8 F	2 h	
FUF	-	148	-	#9 M	Promptly	Helander *et al.*, 2016 [40]
OCF	Snorting	-	Immediate loss of consciousness	N=3 (M)	-	Allibe *et al.*, 2019 [41]

**Abbreviations**: M: male; F: female. ACRYLF Acrylfentanyl; FUF: Furanylfentanyl; OCF: Ocfentanyl. CNS: central nervous system, GCS: Glasgow coma scale.

## LIST OF ABBREVIATIONS

FENS: Fentanyl Derivatives
Fentalogs: Fentanyl Analogs
Fentanyl: N-(1-(2-feniletil)-4-piperidinil)-N-fenil-propanammide
Acrylfentanyl: N-Phenyl-N-[1-(2-phenylethyl)piperidin-4-yl]prop-2-enamide
Furanylfentanyl: N-Phenyl-N-[1-(2-phenylethyl)piperidin-4-yl]furan-2-carboxamide
Ocfentanyl: N-(2-Fluorophenyl)-2-methoxy-N-[1-(2-phenylethyl)piperidin-4-yl]acetamide
Naloxone: (4R,4aS,7aR,12bS)-4a,9-dihydroxy-3-prop-2-enyl-2,4,5,6,7a,13-hexahydro-1H-4,12-methanobenzofuro[3,2-e]isoquinolin-7-one
